# Motivational determinants of athletes’ self-realisation depending on their professional qualification

**DOI:** 10.1186/s40359-024-01895-3

**Published:** 2024-07-31

**Authors:** Yuliya Krasmik, Olga Aimaganbetova, Tatiana Iancheva, Serik Zhantikeyev, Eugeniya Lashkova, Aidos Makhmutov, Bolat Rakhmalin

**Affiliations:** 1 Department of General and Applied Psychology, Al-Farabi Kazakh National University, al- Farabi Ave, 71, Almaty, 050040 Kazakhstan; 2 Department of Psychology, Pedagogy and Sociology, National Sports Academy “Vasil Levski”, Student City, Sofia 1700 Bulgaria; 3 Department of Social Sciences, Humanities and Language Disciplines, Turan-Astana University, Dukenuly street, 29, Astana, 010000 Kazakhstan; 4 Department of Psychotherapy, Center for Clinical Psychology and Psychotherapy Be Alive, Makatayev street, 131, Almaty, 050000 Kazakhstan; 5 Department of Boxing, Weightlifting and Martial Arts, Kazakh Academy of Sport and Turism, Abay Ave, 85, Almaty, 050022 Kazakhstan

**Keywords:** Athletes, Professional self-realization, Resilience, Self-realization, Sports motivation

## Abstract

**Background:**

In the contemporary era, there is a growing emphasis on understanding the self-realization of personality, encompassing individual-psychological traits, abilities, knowledge, skills, and motivation, extensively studied in psychology. Notably, limited attention has been given to exploring motivational determinants influencing athlete self-realization based on their professional qualifications. This gap is particularly pertinent in Kazakhstan, where significant efforts are directed towards athlete preparation.

**Methods:**

Employing a systemic approach by Bagadirova and Kudinov, the study views athlete self-realization as a multi-level phenomenon with interconnected components. A total of 180 athletes participated, including 60 professionals (average age 23.9), 60 amateurs (average age 31.1), and 60 Paralympians (average age 24.4). The methodological toolkit comprises four diagnostic techniques: E.A. Kalinin’s “Motives of Sports Activities” (MSA) questionnaire, S. Maddi’s resilience test adapted by D.A. Leontiev and E.I. Rasskazova, and S.I. Kudinov’s multidimensional questionnaire on self-realization.

**Results:**

Significant differences in motivational determinants and self-realization attitudes emerge across athlete groups based on their sports qualifications. Noteworthy aspects include declining in motivation among professional athletes of masters of sports and masters of sports of international class, and rigidity in life resilience among Paralympic athletes. Overall, self-realization levels are not high, with extremely low levels of professional self-realization in all three athlete categories.

**Conclusions:**

The study’s scientific novelty lies in systematically organizing knowledge on fundamental motivational determinants impacting athlete self-realization according to professional qualifications. This research addresses the acute shortage of psychological studies in sports psychology for Kazakhstan, which is still in its early stages. The findings underscore the need for targeted interventions by coaches and sports psychologists to enhance motivation levels, especially among elite athletes, and foster resilience, particularly in Paralympic athletes, ultimately advancing the field in the country.

**Supplementary Information:**

The online version contains supplementary material available at 10.1186/s40359-024-01895-3.

## Introduction

Professional sports, associated with the notion of success and achieving high results, require resources from athletes related to the display of physical and mental strength, control over outcomes, high involvement, and motivation. Ultimately, this presupposes a relatively high level of awareness of self-realization strategies. Sporting achievements ensure the continuous improvement of the sporting activity itself, reinforce athletes’ motivation, and indicate new paths for the personal development of the athlete. Consequently, sports activities possess significant potential for researching and analysing the psychological mechanisms of personal self-realization.

The completeness of athletes’ self-realization depends on the degree of mobilization of personal resources, primarily motivational [1, 2], which act as long-term regulators of athletes’ activities [1], as well as the dominance of certain self-realization strategies [3]. Self-realization strategies and sports motivation are two psychological components that come into play when discussing athletes’ achievement of high results. Increasing demands for physical fitness, endurance, intense competition, a growing flow of information that needs to be perceived, processed, and applied quickly, lead to an exhaustion of their motivational resources [4]. This can adversely affect athletes’ self-esteem, lead to a loss of motivation for success and ultimately result in the athlete leaving high-performance sports [5, 6].

Thus, motivation is a key determinant that leads to success in sports; it is essential for the development and execution of athletic skills. It is what drives the athlete to successfully acquire new skills through long and intense training sessions.

Studying the motivational determinants of self-realization in professional athletes has gained acute necessity and significance in modern Kazakhstan. In recent years, Kazakh athletes have rapidly been losing their positions in international competitions.

As a result, the country’s sports leadership has placed on the agenda issues related to the preparation and retraining of athletes for international competitions. Approaches and criteria for preparing athletes to achieve high results have been reviewed, recognizing that these are influenced not only by physical and sports training but also by psychological factors. This has led to the actualization of motivational issues for athletes, which have become central in sports psychology today [7–9].

In this regard, the process of intensified training of specialists in sports psychology has actively begun. These specialists, working collaboratively with coaches, will be responsible for creating a motivation and developing successful self-realization strategies for athletes to achieve high results in international competitions.

## Literature review

### Motivation

Motivation should be regarded as the foundation of athletic activity and a leading component of the psychological preparation of the athlete, a crucial condition for the psycho-pedagogical interaction between the coach and the athlete in forming a positive and high motivation for achieving sports results. The athlete’s motivation largely determines the high level of physical, technical, tactical, and psychological preparedness, and consequently, readiness for competitions [10].

In sports psychology, considerable attention is devoted to the study of motivational processes and their influence on overall sports performance [10–13], self-confidence [14, 15], the intention to continue doing sports [16], and self-determination [17, 18]. Additionally, differences related to gender and the level of self-realization were also investigated [10, 19].

The study of the relationship between sports motivation and the efforts exerted has been conducted within the framework of the theory of self-efficacy [16], self-determination theory [20–22], goal achievement theory [23, 24], personality systems interaction theory [25], as well as the theory of deliberate practice in sports and the theory of achievement motivation as expected value [26, 27]. The coronavirus pandemic has led to increased level of interest in research on the motivation of both professional athletes [28–30] and amateur athletes [31–34]. Much attention has been paid to research on the motivation of Paralympians during the coronavirus pandemic [35, 36].

Achievement goal orientation theory (AGT) is one of the leading concepts investigating the individual motivation of athletes. AGT is based on the concept that people will be motivated to achieve success through demonstrating their abilities (competences) – competence can be interpreted as mastering techniques and strategies to enhance individual skill or superiority over other participants [37].

Recent research claims that various achievement goals stimulate varied motivational approaches to exercise [38–40]. A various of studies have shown that athletes achieve higher results after reaching a certain level of goals [41]. However, athletes who achieve high results in the certain circumstances may show suboptimal results [42].

Self-determination theory is a multidimensional concept of motivation based on the idea that there are three types of motivation belonging to the continuum of self-determination. It demonstrates what extent of human behavior is carried out intentionally and autonomously and corresponds to his interests and values [43–45].

In a meta-review within the framework of self-determination theory (SDT), it is noted that, applied to sports, internal motivation and various forms of external motivation have different impacts on athletes’ experiences, well-being, functioning, and performance [46]. A significant body of empirical work has demonstrated the advantages associated with the predominance of autonomous forms of sports motivation: autonomous motivation in sports participation is positively associated with aspects such as persistence, better outcomes, goal-directedness, adaptive coping, increased vitality, and well-being [47, 48]. Autonomous motivation leads to increased level of resilience [49], commitment [50], persistence [51], intention to continue the sports practice [51] and performance [52].

Studies utilizing SDT as a theoretical foundation to examine the relationship between “motivation-performance” have shown that autonomous sports motivation serves as a predictor of objective high performance [53].

Motivation in sports possesses a dynamic nature, with different motivational components dominating at various stages of professional development. Puni [54] described the dynamics of the development of motives in sports activities, linking them to different stages of sports engagement. Motives at the initial stage of sports engagement include the need for movement, the necessity of training, and other motives. Further stages of sports engagement involve moral motives, self-assertion motives, aesthetic motives, and well-being motives. Motives at the stage of highest sports mastery include the key motive of achieving success, social motivation, and material motivation.

Czech researchers in sports psychology, Goshek et al. [55], demonstrated the dynamics of motivational development in sports activities at different stages. They identified four main stages: (1) generalization of motives, (2) differentiation of motives, (3) stabilization of motives, and (4) involution of the athlete’s motivational structure. Thus, in elite sports, the basis of athlete motivation is the desire to overcome the discrepancy between performance standards and their preparedness (both physical and mental).

The dynamics of sports motivation throughout the athletic career of representatives of various sports (volleyball players, rowers) were studied by Ilina [56]. The research revealed a dynamic expression of specific motives over four stages of the athletic career, characterized by unevenness and heterochrony. The peak motivation is reached during the stage of athletic improvement, where the athlete’s motivational structure is most consolidated. Connections between motives and achievement levels were found at all stages of the athletic career.

An athlete’s activity is polymotivated and dynamic; it is not determined solely by one motive. The athlete’s motives undergo age-related changes, changes associated with the growth of their qualifications, and also in connection with their emotional state.

### Hardiness

In some cases, social factors can have a negative impact on athletes, lead athletes to confront significant stress processes such as struggle, self-sacrifice, overcoming difficulties, competition, evaluation, the risk of injury, and defeat [57, 58]. As a result, athletes develop a sufficiently high level of hardiness throughout their sporting careers. *hardiness* has garnered increasing interest as a research subject in the last decade in the psychology of high-performance sports [59, 60].

In the studies, a large number of variables have been identified that help athletes maintain hardiness, including social support, motivation, confidence, and concentration [61]. The rich interconnections obtained indicate the high significance of self-determination motivation in shaping the level of hardiness in athletes of various specializations [62]. In our study, hardiness is understood as the creation of motivation for transformative coping, which implies openness to everything new and the athlete’s readiness to act actively in the stressful situation of preparation for competitions and their performance [63]. The phenomenon of hardiness is a general measure of a person’s mental health and is considered a psychological mediator that contributes to coping with stress and preserving the potential for self-realization in challenging life situations, forming the basis for the self-realization and success of athletes [64, 65]. The notion of hardiness is often associated with overcoming injuries and the process of athletes recovering [66, 67]. Alongside motivational resources, Leontiev considers hardiness and a propensity for risk as resources for self-regulation, which ensure the realization of personal potential [68].

Subsequent studies have shown that the level of hardiness is associated with emotional stability, stress tolerance, adaptability [69], personal self-realization, pessimism/optimism, locus of control, creativity, and motivation [64]. However, the relationships between hardiness indicators and motivation in groups of successful and unstable athletes are not straightforward.

### Self-realization

In sports psychology, the issue of self-realization is addressed in works both by foreign authors [70–76] and by Russian sports psychologists [77–83]. In most works, self-realization is understood as the athlete’s realization of their potential in achieving results, through the improvement of skills and self-expression in sports activities.

In general, the process of self-realization is a time-spanning continuum of realizing one’s potential from a neutral (negative) sphere to a positive one. This process has a heterogeneous (individual components of the mental sphere vary over time) but homogeneous nature, i.e., positive dynamics of one component entail positive dynamics of others, creating a unified structure of the self-realization process. The foundation of self-realization lies in the results of constant self-improvement of the individual. However, self-realization also depends on the confluence of external and internal circumstances, which are not always predictable.

There are some methodological difficulties in studying the process of self-realization associated with terminological ambiguity since “self-realization” is a concept closely related to the description of self-regulation processes and self-organization of activities, self-determination, personal potential, or subjectivity, which, in turn, reflect the substantive-action characteristic of human activity. An important component permeating these synonymous concepts is motivation, its qualitative features, which contribute to achieving the desired outcome [68, 84–87]. In empirical research aimed at studying self-realization, it is quite common to see its manifestation through external objective categories: achieving a certain level of sports qualification [3, 82, 88, 89] and winning high-level competitions [2, 90]. Subjective aspects of self-realization, related to satisfaction with one’s sports career [1, 74] and self-realization strategies [3, 90], have been studied to a lesser extent.

In view of the aforementioned, the objective of the research was to study the motivational profile and characteristics of athletes’ self-realization, taking into account their level of sports qualifications.

## Methodology, materials, and methods

### Research design

**Fig. 1:**
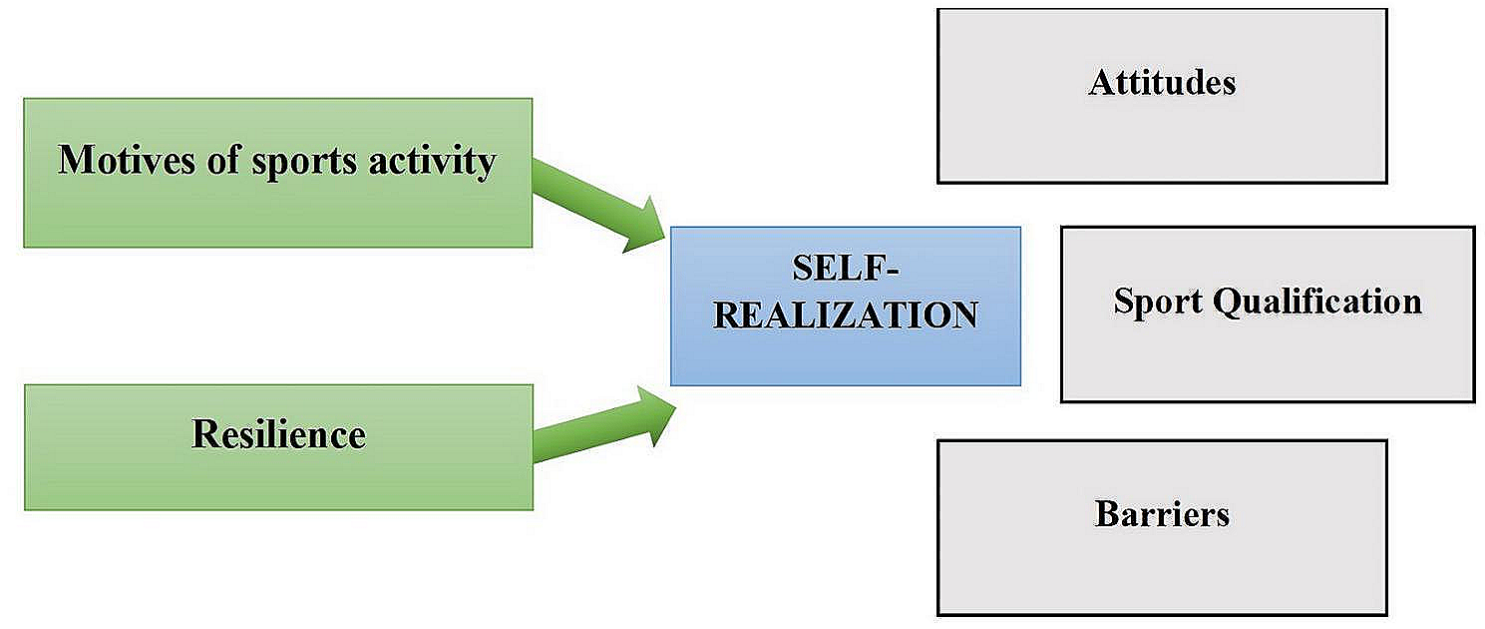
Theoretical Foundations of the Study of Motivational Determinants of Athletes’ Self-Realization

Based on the above, it is necessary to conduct a detailed study of the motivational determinants and components of resilience, the specificity and quality of which ensure the self-realization of athletes in sports and the achievement of high athletic performance by them. In this regard, we are investigating the structure of sports motivation, resilience, involvement in sports activities, readiness for risk, self-realization attitudes, and barriers that contribute to or hinder the self-realization of athletes. The theoretical foundations of the study are presented in Fig. 1.

Within the scope of our research, our aim is to correlate different approaches of motivation and self-determination of athletes. STD, in its main points, closely resembles Leontiev’s model of personal potential, which includes three substructures: the potential for self-determination (choice of need), the potential for achievement (motivation), and the potential for persistence (hardiness) [68]. Their collaborative work serves as a tool, an instrument of personality, if it wants to act based on its own motivation rather than under the influence of internal impulses and external pressure. However, in our view, Leontiev’s approach does not overcome the opposition between external and internal motivation, and the predominance of external motivation is understood as the influence of “alien” motivation [68]. Whereas in the approach of Deci and Ryan [43, 44], this opposition is overcome, and motivation is considered as a continuum, where the contribution of external motivation can positively influence sports achievements.

Building on the work of Gordashnikov and Osin [91] and the self-determination theory of Ryan and Deci [44], we have divided the studied motives and needs into two groups: internal motives and external motives. Internal motivation includes motives such as the achievement motive, struggle motive, self-improvement motive. External motivation includes motives such as communication and encouragement.

We consider self-realization as a multidimensional process of implementing the athlete’s personal potential in achieving results. It encompasses two aspects: the objective aspect - obtaining sports qualifications, winning competitions (achieving athletic success), and the subjective aspect - satisfaction with self-realization in sports, the presence of barriers to self-realization.

*hardiness* is viewed by us, on the one hand, as motivation for coping [63] with emerging sports difficulties, and on the other hand, as a psychological mediator [64, 65], a resource for self-regulation [92] ensuring the preservation of the athlete’s potential for self-realization in sports activities.

### Subjects

Research Base: Academy of Sports in Almaty, Arm-wrestling Federation of Kazakhstan, Weightlifting Federation of Kazakhstan, Powerlifting Federation APF, Paralympic Sports Federation in Kokshetau, Shymkent, Taraz and Sports club for people with disabilities “Mergen”.

The study involved 180 athletes: 60 professional athletes, 60 amateur athletes, and 60 Paralympic athletes (see Table 1).

The gender composition is fairly balanced – 96 men and 84 women. The average age of men is 26.5 years, and the average age of women is 25.9 years.

Among the athletes, there are representatives of various sports: running, triathlon, powerlifting, bodybuilding, judo, rowing, volleyball, karate, football, artistic gymnastics, kudo, athletics, table tennis, arm-wrestling, and others.

**Table 1:** Demographic characteristics of the sample

	Gender	Quantity	Age	Experience in sports	Level of sports qualification
Professional athletes	Men	28	24.4	11.8	MS-9, CMS-11, Н-8
	Women	32	23.4	11.3	MS-16, CMS-8, Н-8
Amateur athletes	Men	31	29.5	7.8	MS-5, CMS-8, Н-18
	Women	29	32.75	9.2	MS-4, CMS-6, Н-19
Paralympic athletes	Men	33	25.0	9.2	MS-7, CMS-12,Н-14
	Women	27	23.8	8.9	MS-9, CMS-4, Н-14

Athletes were divided into 3 groups: professionals (active athletes engage solely in sports activities and receive a salary for it), amateurs (non-professional athletes who engage in sports for their pleasure and to maintain fitness), and Paralympians (athletes with diagnoses of pituitary dwarfism, slirdomutism, the absence of lower extremities and paralysis of the lower extremities). Within each group, there was further division into subgroups based on sports qualification: Master of Sport (МS), Candidate for Master of Sport (CМS), and N (athletes without qualification). In the amateur athletes’ group, only 2 subgroups were identified: athletes with qualification and those without it.

In Kazakhstan, the assignment of sports titles, ranks, and qualification categories occurs in accordance with legislation. The sports title “Candidate for Master of Sports” is awarded for participating in competitions as part of a team that has taken 2nd − 5th place at the Adult Championship, the Republic of Kazakhstan Cup (including open competitions), the Republic of Kazakhstan Spartakiade; 2nd − 4th place in the Youth, 1st Youth Championship (senior age group), 1st − 2nd place in the Republic of Kazakhstan Schoolchildren’s Spartakiade; achieving 12 victories over Candidates for Master of Sports within a year or 8 victories over Candidates for Master of Sports and 8 victories over athletes with a 1st sports rank.

The sports title “Master of Sports of the Republic of Kazakhstan” is awarded to an athlete who has entered the team that took 1st place in team competitions at the Republic of Kazakhstan Championship, the Republic of Kazakhstan Spartakiade, or the Republic of Kazakhstan Cup; 1st − 3rd place in individual or 1st − 2nd place in doubles competitions (men’s, women’s, mixed) - at the Individual-Team Championship of the Republic of Kazakhstan, the Republic of Kazakhstan Spartakiade; 1st − 2nd place in individual competitions, or 1st place in doubles competitions at the Youth Championship of the Republic of Kazakhstan.

The level of sports qualification was confirmed by the presence of titles “Master of Sports of International Class” and “Master of Sports”, combined into one group (MS, 50 people), and “Candidate for Master of Sports” (CMS, 49 people). The third group consisted of athletes with ranks 1–3 and those without sports qualification (N, 81 people).

Research in Almaty and Shymkent was conducted with the personal presence of the researcher. In Taraz and Kokshetau, the research was conducted in an online format, and significant assistance in recruiting athletes for the study was provided by the leaders of sports federations. Some difficulties arose in recruiting the third group of athletes - Paralympians, as they reacted quite emotionally to the research. Significant efforts and close collaboration with the leadership of the federation and the sports club “Mergen” were required to involve them in the study.

### Ethical issues

The study was conducted anonymously, and the collected data were used exclusively for scientific purposes and only in aggregated form. Additionally, they are stored in a secure and protected location. All participants provided informed consent to participate in the research and were informed about how the collected data would be utilized.

### Research limitations

The study employs methodologies that have been regularly and over an extended period used in many CIS countries for conducting research in sports psychology. These methodologies provide reliable results and were adapted by the author for the Kazakhstani sample in 2022.

We acknowledge that depending on the type of sport, there may be some variability in the motives that act as predictors of athletes’ self-realization.

Our sample of amateur athletes consists of people of an older group, who are on average 7–9 years older than the groups of professional athletes and Paralympians. It’s connected to the peculiarities of our sample, which includes the people whose purpose to come to sports was to bring their figures in order and maintain their health, as well as professional athletes who completed their careers and retrained in another sport, for example weightlifters switching to powerlifting, or gymnastics switching to acrobatics.

Also, the limitations of the study include the fact that the group of Paralympic athletes was quite heterogeneous in terms of health abilities.

### Instruments

Psychodiagnostic measurements were conducted using four selected diagnostic methods:

1. *Questionnaire Motives of Sports Activities (*MSA Kalinin, 1974*)* – includes 50 questions, aimed at studying 5 motives: achievement (α = 0.873), struggle (α = 0.885), self-improvement (α = 0.881), communication (α = 0.888), and encouragement (α = 0.901). Adapted for the Kazakhstani sample by author Krasmik in 2022, Cronbach’s Alpha α = 0.966 [62]. The results of the methodology were calculated based on the provided scales according to the methodology key. For example, “achievement” includes statements such as “Feeling confident in achieving the goal,” “Passion and dedication to work,” “Hope for achieving very high sports results,” and others. The rating scale for the expression of individual motives in sports activities consists of 4 levels: elevated motivation − 30 to 40 points; optimal motivation − 28 to 33 points; lowered motivation − 19 to 27 points, and low motivation − 10 to 18 points. When developing the scale for assessing the level of motives, the author relied on the Yerkes-Dodson law, which suggests that the best results are achieved at moderate motivation intensity.
2. *S. Maddi’s Hardiness Test (*adaptation by Leontiev, Rasskazova) – the methodology consists of 45 questions, measuring 4 indicators: challenge, control, commitment, and overall hardiness level [93]. This adaptation is built based on the third version of the resilience questionnaire, The Personal Views Survey III-R. The original three-scale structure was retained: challenge, control, commitment. The results of the methodology were calculated based on the provided scales according to the methodology key.
3. *S.I. Kudinov’s Multidimensional Personality Self-Realization Questionnaire* [94] – the questionnaire allows identifying the specificity of personal self-realization and includes 101 questions. The methodology contains 16 bipolar scales characterizing opposite characteristics manifested in activities, behavior, and communication: 1 – meaningfulness of self-realization goals-values; 2 – awareness of goals-values (value-target component); 3 – energy; 4 – lack of energy (dynamic component); 5 – optimism; 6 – pessimism (emotional component); 7 – internality; 8 – externality (organizational component); 9 – sociocentrism; 10 – egocentrism (motivational component); 11 – creativity; 12 – conservatism (cognitive component); 13 – constructiveness; 14 – destructiveness (prognostic component); 15 – social barriers to self-realization; 16 – personal barriers to self-realization (competence-personal component) [94]. The results of the methodology were calculated based on the provided scales according to the methodology key. For example, the internality scale includes statements such as “You easily manage social work due to good self-control,” “You are absolutely sure that personal self-improvement depends solely on the individual,” and others. The methodology also allows assessing the level of self-realization, which is measured on a scale ranging from irrational-inert-adaptive-harmonious-intense. At the final stage, the overall level of self-realization and the predominance of one of the forms of personal, social, or professional self-realization are calculated using formulas.

At the end of the study, a short questionnaire consisting of 7 questions was proposed: gender, age, sport discipline, sports qualification, years in sports, group (professionals, amateurs, Paralympians), and “main goal related to achievements in sports.”

These diagnostic techniques did not require an agreement, they can be used in research without any copyright restrictions.

### Data analysis

In this work, comparative analysis was chosen as the main method for comparing two or more groups, identifying commonalities and differences with the aim of classification, and understanding the specific features of each group. For comparing the data of two groups, the Mann-Whitney’s U-criterion was selected for two independent samples, the most well-known and widely used non-parametric test for comparing two independent samples. To compare three groups, the Kruskal-Wallis H-criterion was used, which is a generalization of the Mann-Whitney U-criterion for three or more independent samples. Statements with a probability of error *p* ≤ 0.05 are considered significant, statements with a probability of error *p* ≤ 0.01 are highly significant, and statements with a probability of error *p* ≤ 0.001 are maximally significant. The presented tables include only significant differences to make them more user-friendly.

Statistical data processing was carried out using the SPSS program, version 26.0.

### Research results

We conducted a comparative analysis of motivation features in each group based on athletes’ qualification levels. The obtained data are presented in Tables 2 and 3.

**Table 2:** Descriptives statistics individual motives among athletes according to the MSD

	Range	Minimum	Maximum	Mean	Std. Deviation
**Professional athletes**					
Achievement	21.0	19.0	40.0	32.49	5.23
Struggle	30.0	10.0	40.0	28.77	6.37
Self-improvement	24.0	16.0	40.0	31.26	5.244
Communication	26.0	10.0	36.0	24.02	5.97
Encouragement	28.0	12.0	40.0	27.47	7.72
**Paralympic athletes**					
Achievement	30.0	10.0	40.0	30.89	7.17
Struggle	30.0	10.0	40.0	27.60	7.02
Self-improvement	30.0	10.0	40.0	31.11	7.25
Communication	27.0	9.0	36.0	23.91	6.93
Encouragement	30.0	10.0	40.0	26.41	8.14
**Amateur athletes**					
Achievement	28.0	12.0	40.0	30.69	6.24
Struggle	30.0	10.0	40.0	27.54	7.31
Self-improvement	30.0	10.0	40.0	29.81	6.85
Communication	27.0	9.0	36.0	22.32	6.99
Encouragement	30.0	10.0	40.0	23.03	7.70

The profile of motives for sports activities showed that professional athletes have an elevated motivation for achievements (M = 32.5). Self-improvement motivation ranks second among professional athletes (M = 31.3), and the motivation of struggle is in third place (M = 28.8), with the levels of these two motives being optimal. The motivation profile of amateur athletes is similar to that of professional athletes but without elevated achievement motivation; achievement (M = 30.7) and self-improvement (M = 29.8) motives are optimal, contributing to maintaining a stable motivation level during the training process (according to the Yerkes–Dodson law). The motivation profile of paralympic athletes indicates that optimal motivation is present for two motives – self-improvement (M = 31.1) and achievements (M = 30.9). The group of paralympic athletes differs in that self-improvement motivation takes the first place.

**Table 3:** Differences in the expression of individual motives among athletes according to the MSD questionnaire depending on the level of sports qualification

**Motives**	**Professional athletes**				**Kruskal-Wallis test**	**Sig.**
	MS	CMS		Н		
Struggle	28.1	26.5		16.1	5.680	0.05
Encouragement	28.2	24.2		17.1	5.718	0.05
	**Paralympic athletes**				**Kruskal-Wallis test**	**Sig.**
	MS	CMS		**Н**		
Encouragement	43.9	26.4		27.3	5.905	0.05
	**Amateur athletes**				**Mann-Whitney U**	**Sig.**
	**MS/CMS**		**Н**			
Struggle	38.8		27.8		163.500	0.05
Communication	39.9		27.5		163.500	0.03
Encouragement	38.8		27.4		178.500	0.05

The presented tables include only significant differences to make them more user-friendly. Statements with a probability of error *p* ≤ 0.05 are considered significant.

The obtained data indicate significant differences in the expression of individual motives among the group of professional athletes depending on the level of sports qualification: the motive of struggle (*p* = 0.05) and the reward motive (*p* = 0.05).

In the group of para-athletes, significant differences in the expression of sports activity motives were identified depending on the level of sports qualification for one motive – the reward motive (H = 5.905; *p* = 0.05). This motive is more developed in the subgroup of sports masters and masters of international class than in the subgroup of candidates for sports masters and the subgroup with no sports qualification.

Significant differences in the expression of individual motives were also found in the group of amateur athletes depending on the level of sports qualification: the motive of struggle (*p* = 0.048), communication motive (*p* = 0.025), and the reward motive (*p* = 0.050). These motives are more pronounced in the subgroup of amateur athletes with sports qualifications.

The overall level of *hardiness* among professional athletes is 78.9 points, among amateur athletes is 77.8 points, and among para-athletes is 71.3 points (see Table 4). This corresponds to an average level of effective coping with stressful situations and indicates a formed motivation for transformational coping during training, competitions, setbacks, and instances of injury.

**Table 4:** Descriptives statistics on hardiness

	Range	Minimum	Maximum	Mean	Std. Deviation
**Paralympic athletes**					
Commitment	41.0	11.0	52.0	34.06	9.06
Control	28.0	15.0	43.0	27.93	6.85
Challenge	17.0	8.0	25.0	16.87	4.46
Overall hardiness level	69.0	43.0	112.0	78.87	17.93
**Paralympic athletes**					
Commitment	54.0	0.0	54.0	29.04	9.61
Control	48.0	0.0	48.0	26.49	9.20
Challenge	30.0	0.0	30.0	15.81	6.25
Overall hardiness level	132.0	0.0	132.0	71.34	23.51
**Amateur athletes**					
Commitment	40.0	10.0	50.0	33.83	9.30
Control	33.0	10.0	43.0	27.72	7.75
Challenge	21.0	5.0	26.0	16.27	5.38
Overall hardiness level	85.0	29.0	114.0	77.83	20.01

The presented tables include only significant differences to make them more user-friendly. Statements with a probability of error *p* ≤ 0.05 are considered significant.

The data presented in Table 5 indicate that significant differences in *hardiness* indicators have been identified based on the qualification of professional athletes: on the commitment scale (*p* = 0.05) – Candidates for Master of Sports (CMS) exhibit greater self-confidence, satisfaction with their sports activities, and perceive the world as generous, providing opportunities for the realization of their potential; on the challenge scale (*p* = 0.017) – CMS athletes tend to actively acquire new knowledge and experience, seek to extract lessons from any life or sports events, whether positive or negative, and have a pronounced willingness to act despite fear and risk in competitions to achieve victory; overall *hardiness* level (*p* = 0.021) – CMS athletes demonstrate a system of beliefs and motivation that prevents the occurrence of high levels of tension during training and competitions, thanks to their *hardiness* coping with anxiety and stress.

**Table 5:** Differences in the expression of hardiness indicators among athletes depending on the level of sports qualification

Indicators of Resilience	Professional athletes			Kruskal-Wallis test	Sig.
	MS	CMS	Н		
commitment	21.5	33.4	21.453	5.991	0.05
challenge	20.02	34.65	22.93	8.117	0.02
hardiness Level	20.83	34.65	21.61	7.718	0.02
	Amateur athletes			Mann-Whitney U	Sig.
	MS/CMS	Н			
challenge	20.4	33.7		113.000	0.04

No significant differences in *hardiness* and its components were found in the Paralympic group.

In the group of amateur athletes, significant differences were identified in one challenge scale (U = 113.000; *p* = 0.042).

The overall level of self-realization in three groups corresponds to the average (professional athletes – 66.2; amateur athletes – 55.6; paralympic athletes – 56.2), adaptive level, indicating moderation and a desire to be not inferior and to excel in sports activities compared to others. However, professional self-realization in sports is extremely low (see Table 6). Among professional athletes and amateur athletes, three self-realization attitudes dominate – creativity, internality, and constructiveness. Among paralympic athletes, the dominant attitudes are internality, creativity, and meaningfulness of goals and values.

**Table 6:** Descriptives statistics on self-realization

	Range	Minimum		Maximum	Mean		Std. Deviation
**Professional athletes**							
Value-target component	22.0	-2.0		20.0	8.04		6.53
Dynamic component	32.0	-7.0		25.0	7.23		7.52
Emotional component	31.0	-2.0		29.0	10.59		7.41
Organizational component	29.0	-1.0		28.0	11.25		7.50
Motivational component	27.0	-15.0		12.0	-2.78		5.87
Cognitive component	27.0	-2.0		25.0	11.04		7.30
Prognostic component	30.0	0.0		30.0	9.97		7.25
Competence-personal component	22.0	-13.0		9.0	-2.06		5.28
Personal self-realization	58.00	3.00		61.00	36.36		14.73
Social self-realization	58.25	-22.75		35.50	9.96		11.76
Professional self-realization	57.50	-5.00		52.50	19.87		12.94
Competence-Personality Component	127.25	7.25		134.50	66.20		31.16
**Paralympic athletes**							
Value-target component	32.0	-10.0	22.0		6.42	6.30	
Dynamic component	34.0	-14.0	20.0		5.22	7.36	
Emotional component	47.0	-25.0	22.0		7.76	7.80	
Organizational component	29.0	-5.0	24.0		10.12	6.50	
Motivational component	31.0	-19.0	12.0		-1.47	5.53	
Cognitive component	29.0	-4.0	25.0		8.87	6.55	
Prognostic component	33.0	-10.0	23.0		7.92	6.83	
Competence-personal component	32.0	-20.0	12.0		-2.84	5.24	
Personal self-realization	57.75	2.25	60.00		31.50	14.11	
Social self-realization	65.50	-32.25	33.25		7.56	13.98	
Professional self-realization	59.50	-3.25	56.25		17.15	14.86	
Competence-Personality Component	135.50	-12.25	123.25		56.21	35.62	
**Amateur athletes**							
Value-target component	27	-7	20		6.02	5.59	
Dynamic component	28	-8	20		4.00	6.83	
Emotional component	36	-14	22		7.22	6.90	
Organizational component	39	-12	27		9.00	8.54	
Motivational component	34	-21	13		-1.64	7.15	
Cognitive component	27	-5	22		8.03	6.49	
Prognostic component	24	-6	18		7.37	6.23	
Competence-personal component	17	-11	6		-2.56	4.36	
Personal self-realization	73.00	-5.75	67.25		28.44	17.13	
Social self-realization	62.50	-13.25	49.25		8.96	12.59	
Professional self-realization	63.00	-11.25	51.75		18.20	17.80	
Competence-Personality Component	181.25	-13.00	168.25		55.61	41.71	

A comparison of the expression of components and types of self-realization depending on the level of athletic qualification among athletes was conducted, and the obtained data are presented in Table 7. The presented tables include only significant differences to make them more user-friendly. Statements with a probability of error *p* ≤ 0.05 are considered significant.

**Table 7:** Differences in the expression of components and types of self-realization depending on the level of sports qualification among athletes were assessed using the multidimensional questionnaire of personality self-realization by S.I. Kudinov

Attitudes	Professional athletes				Kruskal-Wallis test	Sig.
	MS	CMS		Н		
Value-target component	21.48	34.30		20.79	7.210	0.03
Dynamic component	23.63	33.75		17.64	8.124	0.02
Organizational component	22.83	33.95		18.36	6.110	0.05
	**Paralympic athletes**				**Kruskal-Wallis test**	**Sig.**
	**MS**	**CMS**		**Н**		
Organizational component	41.53	30.59		27.81	6.066	0.05
Prognostic component	40.59	25.78		30.77	5.908	0.05
Professional self-realization	40.19	25.88		30.94	5.986	0.05
	**Amateur athletes**				**Mann-Whitney U**	**Sig.**
	**MS/CMS**		**Н**			
Competence-Personality Component	22.75		31.85		185.000	0.05

The data presented in Table 7 indicate that significant differences in the expression of self-realization components have been identified based on the qualifications of professional athletes: value-target component (*p* = 0.027). Athletes with the title of Candidate for Master of Sport (CMS) exhibit a pronounced orientation toward team goals and a sense of camaraderie; dynamic component (*p* = 0.017) – CMS athletes demonstrate a high level of activity and energy; organizational component (*p* = 0.052) – CMS athletes exhibit a high level of self-control and self-organization during the preparation for competitions.

Significant differences in the expression of self-realization components were also identified based on the qualification of para-athletes: organizational component (*p* = 0.048) athletes with the titles of Master of Sports (MS) and International Master of Sports (IMS) exhibit a high level of self-control and self-organization during the preparation for competitions; prognostic component (*p* = 0.050), athletes with the titles of Master of Sports (MS) and International Master of Sports (MSMK) show a high level of satisfaction with the training process and sports activities, achieving high success in mastering new milestones; professional self-realization (*p* = 0.049), athletes with the titles of Master of Sports (MS) and International Master of Sports (MSMK) demonstrate a higher level of achieving significant results in sports activities.

In the group of amateur athletes, significant differences in the expression of self-realization components were identified depending on the qualification of professional athletes, only for one aspect – the competence-personality component (U = 185.000; *p* = 0.05). This component holds greater significance for non-qualified amateur athletes. For them, personal barriers, inhibition, lack of confidence, sensitivity, and resistance to criticism more significantly hinder the achievement of their goals.

## Discussion

### Motivational profile

During the process of professional development, self-realization of athletic potential, and the attainment of high levels of sports qualification, the formation and development of both internal and external motives take place.

The significance of motives such as achievement, struggle, self-improvement for professional athletes corresponds to the model of motivation for high-class athletes, according to Sopov [95]. Our data, overall, align with the findings of Arinchina’s research [96], which demonstrated that professional athletes experienced significantly greater needs for achievement, the pursuit of these achievements, self-improvement, and material rewards for their accomplishments. Similarly, in the study by Germanov et al. [97], regardless of the type of sport, the motivation for achievement is at the top of the hierarchy for professional athletes. A high level of motivation signals the choice of an activity in accordance with individual needs and preferences [98]. Motivation for success is the most positive component of motivation in sports. An athlete realizes that athletic performance depends on his hard work and efforts. This kind of athlete is motivated by hard work (which he puts into training and competitions), progress, training and development of abilities. The athlete has strong internal control, he is self-motivated and goal-oriented. Also he is motivated by the desire for success and the opportunity to influence other sports participants [99].

It should be noted that as the athletic potential is realized in all three groups, external motivation evolves, encompassing encouragement. Svilina and Safiullin [100] also points to the predominance of the material component in the motivational framework of modern athletes. It is important to note that excessive striving for external rewards is one of the risk factors for emotional burnout of athletes [101]. The internal motivation for competition, in the process of achieving a high level of sports qualification, is formed only in two groups – professional athletes (struggle) and amateur athletes (struggle). It is noteworthy that internal motivation among Paralympic athletes does not undergo development during the process of self-realization and improvement in sports qualification; rather, only external motivation (encouragement) shows progression.

To some extent, a similar dynamic of changes in motives is shown in the study by Castro-Sánchez et al. [102], which demonstrated that task orientation prevails among professional athletes, while ego-goal orientation is predominant among amateurs. As the level increases among amateur athletes, goal-oriented ego becomes more significant.

Regarding the data on the significance of motives in the group of amateur athletes, similar findings were obtained in the study by Bochaver et al. [103], which highlighted the importance of motives related to appearance, physical self-improvement, and health for amateur athletes.

The formation of motives among Paralympic athletes is associated with the growth of sports qualification: the higher their qualification, the more pronounced is the development of orientation towards reward and approval. These results align with the findings of the dissertation research by Shamych [104], which demonstrated that material incentives (salary, uniforms, bonuses, etc.) are essential subjective stimuli for engaging in sports among Paralympic athletes with the highest level of performance.

For Paralympic athletes, a high significance of a collective orientation and their inclination towards social interaction is evident. Research conducted on visually impaired athletes has shown that achieving social identity and regulating social relationships play a crucial role in their motivation for sports activities [105]. Thus, involving individuals with limited abilities in Paralympic sports significantly enhances their capacity for comprehensive personal development and complete integration into society [106–108].

Torralba Jordán et al. [109] determined that the most significant motives for engaging in sports were associated with social issues and overcoming them. A study by Van Biesen and Morbee [35] identified only three motivational types in the group of Paralympic athletes. In the first profile, all types of motivation were present, but amotivation dominated. In the second profile, the autonomous type of motivation was most pronounced, albeit to a minor extent. In the third profile, the controlled type of motivation was dominant.

At the same time, some researchers emphasize the need for targeted assistance to Paralympic athletes in managing motivation and developing the necessary psychosocial skills [110–113].

### Hardiness

The data obtained from the hardiness e test show that the motivation for coping, manifested in commitment, challenge, and control, also changes as athletes self-realize and achieve a certain level of sports qualification. In the group of professional athletes, differences were identified, revealing that components of existential courage such as commitment, challenge, and overall hardiness are more developed among athletes with Candidate for Master of Sports (CMS) titles. It is at this level of self-realization in professional athletes that motivation for coping unfolds to the maximum. However, among Masters of Sports (MS) and Masters of Sports of International Class (MSIC), hardiness levels decrease significantly, indicating that, for some reasons, professional athletes may shift from internal self-regulation and motivation for coping to external, extrinsic regulation, or even experience a lack of regulation. This could suggest a depletion of motivation for coping.

Our data do not align with the results of Gould et al. [114], Fletcher and Sarkar [115], who demonstrated that athletes at the highest level exhibit a greater ability to cope with failures, stress, and adversities.

A high level of challenge has a decisive impact on competitive performances [116–118]. One of the comparative studies of the term of hardiness showed and proved that that excessive levels of control exhaust parachutists. The research was directed on the representatives of parachuting and yoga sports. At the same time, representatives of yoga with an average level of control have significantly higher indicators of dispositional viability and hardiness [119].

In the group of amateur athletes, a different picture emerges, where one of the indicators of hardiness – the challenge – is predominant among athletes without sports qualifications. In other hardiness indicators, the experience gained in achieving sports qualifications does not exert a significant influence on the development of hardiness in the group of amateur athletes.

The hardiness level of Paralympic athletes remains consistently low regardless of the improvement of their sports skills and the achievement of new levels of sports qualification. This may indicate the demotivation of the athletes, as victories, accomplishments, and results do not enhance their engagement in sports activities, fail to bring satisfaction, and do not instill confidence in the chosen path. Our results do not coincide with the research data of Tretyakova et al. [120], according to which the overall level of hardiness significantly differs depending on the level of qualification of athletes. The fact that no significant differences were found within the group of Paralympic athletes requires further investigation and may be associated with the specific circumstances of Paralympic athletes in Kazakhstan. Despite their achievements in the Paralympic Games, they receive little support and recognition.

### Self-realization

In studies of athletes’ self-realization, it is typically examined not based on psychological indicators, but rather on the presence of specific sports qualifications or by considering athletes’ participation in international competitions and their performance in them [121, 122]. The obtained data generally align with the conclusions of Afanasyev et al. [123] regarding athletes’ insufficient life satisfaction, the absence of trustful relationships within the team and with the coach. It can be assumed that there is a crisis in the approaches to athletes’ training and its reevaluation, which is expressed, on the one hand, in the reduction of self-realization among athletes and, on the other hand, in accompanying changes.

The results of the study on the self-realization of Ukrainian Paralympians by sports psychologists show outcomes different from ours: Paralympians tend to have satisfaction with their own sports career, a relatively quick establishment of new sports goals, and a clear vision of their sports future. A significant portion of them does not consider their current sports achievements as the ultimate possibilities, seeing the potential for further sports improvement [124].

In the group of professional athletes with the title of Candidate for Master of Sports (CMS), three components of self-realization – value-oriented, dynamic, and organizational components – are significantly more developed. This indicates that CMS athletes have a clear and meaningful strategy for realizing their athletic potential; they are more goal-oriented and persistent in achieving sports results, work on mistakes, and develop self-control. It is noteworthy that in professional athletes with higher qualifications (Master of Sports/Master of Sports of International Class), the level of expression of these self-realization components is significantly lower than in CMS athletes. This may be indicative of a kind of “glass ceiling” in sports, where high-level athletes have nowhere to grow, and there is no opportunity for further development. Our data do not coincide with the findings of Sergeeva [82], which demonstrated that the self-realization assessment of athletes consistently increases along with the level of proficiency: the lowest among non-qualified athletes, an intermediate score among those with Candidate for Master of Sports (CMS) qualification, and the highest score among Masters of Sports (MS). It is worth noting that Sergeeva [82] study evaluated the level of self-realization not through diagnostic methods but based on the performance of athletes.

In a group of amateur athletes, the difference in the development of self-realization components depends on achieving a certain level of qualification and is insignificant. Significant differences have been identified only in one aspect – the competence-personality component. This component is more significant for non-qualified amateur athletes, as personal barriers such as rigidity, lack of self-confidence, sensitivity, and resistance to criticism hinder them more when achieving their goals. The obtained result is quite logical; attaining a higher level of sports qualification enables athletes to overcome self-doubt, makes them more receptive to criticism, and they react less emotionally to remarks during training, understanding that the coach is correcting technique rather than making personal accusations.

For Paralympic athletes, sports become a vital source of self-realization and existence, a crucial means of post-traumatic recovery, adaptation, integration, and inclusion in social life. Close interaction with able-bodied athletes opens up opportunities for establishing new friendships, increasing life satisfaction, and balancing the consequences of disability [125].

Since disability limits the ability to perform physical exercises, achieving high results requires Paralympic athletes to adhere to clearly defined rules of sports activities, exert significant efforts, and comply with a wide range of restrictions and prohibitions that affect their self-realization.

In the group of Paralympic athletes, an entirely different picture is observed, unlike the two previous groups of athletes. Significant differences have been obtained, showing that the development of self-realization components is more pronounced the higher their level of sports qualification. Masters of Sports (MS) and Masters of Sports of International Class (MSIC) are characterized by higher values in terms of the level of professional self-realization and the development of prognostic and organizational components.

The research conducted by Shamych [104] revealed similar results: considering the varying age and level of sporting achievements among Paralympians, a significant dispersion of indicators was identified in responses regarding whether they have already reached the pinnacle of success in sports. Approximately one-third of them believe that they have not yet reached the peak of their achievements or have only partially achieved it. Slightly more than a quarter are convinced that they have almost or completely reached such a pinnacle. It is important to note that self-realization was comprehensively assessed by the authors based on six internal indicators (enjoyment of training, physical exertion; moral satisfaction from victories in competitions; the opportunity for personal self-realization; the opportunity to represent their country, club, city in competitions; communication with friends, acquaintances; the opportunity to correct or compensate for health deficiencies) and four external indicators (the opportunity or prospect of foreign trips; material incentives – salary, food, awards, etc.; gaining experience, making acquaintances, etc., which can help to settle down in later life; the opportunity to be a full-fledged member of society). A comprehensive approach to the issue of self-realization shows that it has ceased to be merely the achievement of high sports results [126] and has become embedded in the context of the athlete’s mental health and well-being [127, 128].

It was found that three parameters – “emotional stability of athletes”, “area of achievement of athletes” and “health and illness of athletes” - are essential and interrelated with both motivations: motivation to achieve success (MAS) and motivation to avoid failure (MAF) [129].

The presented results require a comprehensive approach when working with the motivation and self-realization of athletes. Concern arises from the decline in motivation levels among high-class athletes, those athletes from whom victories are expected in international competitions. Low self-realization indicators, on the one hand, indicate that athletes set vague and undefined goals for themselves, and on the other hand, it reflects the lack of state support and the need to independently seek financing for participation in competitions. Thus, efforts to improve the efficiency and performance of Kazakhstani athletes in international competitions will be associated with numerous reforms. These reforms primarily involve the implementation of new, modern methodological principles for preparing athletes for competitions, aimed at fostering and sustaining motivation at every stage of sports activity, shaping clear and adequate prospects for self-realization in the chosen sport, and, of course, financial support from the state.

## Conclusion

In our study, motivational predictors of athletes’ self-realization were studied depending on the level of sports qualifications. We have found that athletes with different levels of athletic qualifications have certain characteristics in sports motivation, hardiness and self-realization.

Professional athletes with higher qualifications (MS) have high motives for struggle and encouragement, however, professional athletes with lower qualifications (CMS) shown higher results in hardiness, commitment and challenge, as well as in some components of self-realization. Professional athletes with higher qualifications (MS) have high motives for struggle and encouragement, however, professional athletes with lower qualifications (CMS) showed higher results in hardiness, commitment and challenge, as well as in some components of self-realization.

Highly qualified Paralympians demonstrate the high importance of the motives of encouragement and professional self-realization. Amateur athletes with high qualifications shown the great importance for them of the motives of struggle, communication and encouragement. Amateur athletes without sports qualifications have a higher level of challenge and high personal barriers to achieving self-realization.

The obtained data allowed to identify aspects that require the attention of coaches and sports psychologists, primarily related to a decrease in motivation among professional athletes of a high classification level and low indicators of hardiness among Paralympic athletes. And at the same time identified the low scores on satisfaction with the subjective components of self-realization in the presence of its high objective indicators.

We urgently need intervention measures to support our athletes in realizing their potential and developing strategies that would contribute to the growth of hardiness and subjective satisfaction with their results, the way their professional careers are developing. In general, the results of the study led us to the need of search for additional variables that hinder the self-realization of athletes when forming a motivational profile corresponding to high-class athletes.

### Electronic supplementary material

Below is the link to the electronic supplementary material.

Supplementary Material 1

## Data Availability

Data will be available from the corresponding author on reasonable request.

## Abbreviations

MS: Master of sports
CMS: Candidate for master of sports
MSIC: Master of sports of international class
