# Supplementary material for: Motivational determinants of athletes’ self-realisation depending on their professional qualification
Source: BMC Psychol. 2024 Jul 31;12:416. doi: 10.1186/s40359-024-01895-3 (PMC11292874; doi:10.1186/s40359-024-01895-3)
Supplement: Supplementary file 1 — Supplementary Material 1 [file 40359_2024_1895_MOESM1_ESM.docx]

**(1) Methodology "Study of motives for sports activities"**

The method was developed by V.I. Tropnikov to find out the degree of importance of various reasons (situations, circumstances) that motivated and encourage the athlete to continue practicing the chosen sport.

*Instructions.*

Try to assess as accurately as possible the reasons (situations, circumstances) that motivated and encourage you to pursue this sport.

You will be given a list of possible reasons that athletes usually give.

First of all, look through the whole list. All of the reasons should be rated from 5 to 1 in terms of their significance and importance for you to continue playing the sport. Reasons that are not important to you are given a score of 1.

**Questionnaire text** (possible reasons).

1. On medical advice.

2. The desire to cultivate determination.

3. To get sports uniforms and equipment.

4. On the advice of parents, relatives.

5. The desire to have a figure with a strongly developed musculature.

6. Because it is a beautiful sport.

7. To have more friends and comrades.

8. Because playing sports allows you to increase your authority at work (place of study).

9. To acquire skills because they will be useful in life.

10. The desire to defend the honor of the team (sports society) at various competitions.

11. To broaden my horizons, world outlook.

12. Because it feels good to be praised and approved by the coach.

13. Because the sports base is close to home (place of study, work).

14. There were no other sections.

15. I am tall, which is valuable for this sport.

16. The desire to get the next sport category.

17. The desire to become a champion of the city, country.

18. The desire to develop mobility and coordination.

19. The desire to develop strength.

20. The purpose of maintaining good health.

21. A desire to cultivate courage and determination.

22. Because athletes are given food stamps.

23. On the advice of friends, comrades.

24. Because it is a sport where you can train individually, independently of others.

25. Desire to have a beautiful slim figure.

26. The desire to be in some kind of collective.

27. Because practicing sports allows you to increase your authority among relatives, friends, comrades.

28. To acquire skills, as they will be useful during military service.

29. The desire to protect the honor of the team (city) at various competitions. 30. The desire to learn more about the lives of outstanding athletes.

31. Because I am pleased when my parents and relatives praise me for my results.

32. From nothing to do (a lot of free time).

33. Watched a movie, TV show about this sport.

34. I consider myself agile and with good coordination, which is valuable for this sport.

35. Desire to become a master of the sport.

36. To have a fun time.

37. A desire to develop endurance.

38. Desire to develop speed qualities.

39. To maintain a constant level of physical development.

40. A desire to develop willpower.

41. Because athletes have the opportunity to receive valuable prizes and gifts.

42. Because of family traditions.

43. Because I like the complex tactical struggle in competitions in this sport.

44. Because I like the beauty and precision of the execution of exercises in this sport.

45. Because I like the collective training.

46. The desire to become a leader, a team captain.

47. To get a specialty, to work in the field of sports in the future.

48. The desire to defend the honor of their country in international competitions. 49. The desire to visit other cities for training camps and competitions.

50. Because I am pleased when my friends and comrades approve and praise me for my achievements.

51. Because this sport is developed at my place of study (work).

52. Because I have been to competitions in this sport.

53. I believe that I have good speed qualities, which is valuable for this sport.

54. I want to become a champion of the country, the world and the Olympic Games.

55. Because it is an emotional sport.

56. Because the competition in this sport is exciting and attractive.

57. The desire to develop agility.

58. To get rid of excess weight, obesity.

59. To develop persistence and perseverance.

60. Because athletes have the opportunity to receive monetary, material support.

61. Because parents played the sport.

62. Because in this sport the growth of own results is most clearly seen.

63. I feel the need for motor activity.

64. A desire to meet famous people and leading athletes.

65. To keep up with my friends and comrades.

66. Because playing this sport helps in various life situations.

67. Because it feels good to feel a sense of accomplishment to my teammates.

68. The desire to visit competitions abroad.

69. Because it makes me feel good to be praised for my accomplishments.

70. Meeting (acquaintance) with famous athletes.

71. Not admitted to other sections.

72. I consider myself physically strong, which is valuable for this sport.

73. I consider myself hardy, which is valuable for this sport.

74. Because playing sports is a form of entertainment for me.

75. Because I like thrills.

76. A desire to develop flexibility.

77. In order not to get sick, to be always healthy.

78. The desire to have a strong character.

79. Because athletes have the opportunity to get a room, an apartment, to improve living conditions.

80. Because the brother (sister) practiced (is practicing) this sport.

81. Because practicing this sport develops the ability to think quickly and accurately.

82. It gives pleasure to experience physical exertion.

83. Because playing sports increases self-esteem.

84. To be more attractive to the female (male) sex.

85. Because it makes me feel good to have a sense of accomplishment to my coach.

86. To fulfill the expectations placed on me by my coach, my parents. 87. I am fascinated by the need to constantly search for better training methods.

88. Because the physical qualities developed by this sport are valued by the people around me.

89. I started practicing by accident.

90. Invited to practice by the coach.

91. I believe that only in this sport I can achieve significant success.

92. I am short in stature, which is valuable in this sport.

93. Because I like to compete just for fun, whether I win or lose.

94. Because I enjoy experiencing the joy of winning.

95. Because playing sports enhances intellectual development.

96. To recover more quickly from an illness.

97. The desire to cultivate stamina and self-control.

98. Because athletes have the opportunity to dress fashionably (sports style).

99. Because all my friends are involved in sports.

100. Because playing sports develops the skills to fight back against bullies.

101. Because I don't get tired physically at work (study).

102. The desire to become a leader among friends and comrades, to be respected by them.

103. Because it feels good to feel physically superior.

104. To quit bad habits, to break with bad company, to distance myself from the street.

105. Because it pleases me to feel a sense of accomplishment in my duty to society.

106. Because I like the presence of relatives, friends, comrades at competitions, who cheer for me and admire my successes.

107. Because it makes me feel good to see athletes on television, on the radio, in newspapers and magazines.

108. I read the advertisement for enrollment in the section.

109. Accidentally performed at a competition in this sport.

**Results processing**

Comparison of the degree of expression in the athlete of a particular

motive or need:

- communication (items 7, 26, 36, 45, 65, 74,99, 102),
- cognition (items 11,30, 64, 87),
- material goods (items. 3, 22, 41, 49, 60, 68, 79, 98),
- development of character and mental qualities (items. 2, 21, 40, 59, 78, 81, 95,
- 97),
- physical perfection (items. 5, 18, 19, 25, 37, 38, 39, 57, 76, 84),
- well-being and health improvement (items. 1, 20, 58, 63, 77, 96, 101, 104), aesthetic pleasure and thrill (paras. 6, 43, 44, 55, 56,
- 75, 82, 93, 94),
- acquisition of skills and knowledge useful for life (items. 9, 28, 47, 66,
- 88, 100),
- need for approval (items 12, 31, 50, 69, 106),
- increasing prestige, desire for glory (pts. 8, 16, 17, 27, 35, 46, 54, 83, 103, 107),
- collectivistic orientation (items 10, 29, 48, 67, 85, 86, 105).

Since the number of items for each motive in the methodology is different, a comparison of the expression of these motives is possible if the sum of scores is divided by the number of items related to each motive, i.e. the arithmetic mean of the expression of each motive is calculated.

According to the answers to items. 4, 13, 14, 23, 24, 32, 33, 42, 52, 52, 61, 70, 71, 80, 89, 90 it is possible to find out specific motives of a given person's coming to sport.

And by the answers to items. 15, 34, 51, 53, 53, 62, 72, 73, 91, 92 - why this particular sport was chosen.

**(2) Motivation for Sports Activity (MSA) by E.A. Kalinin**

*Instruction.* Read the statement and select how often you experience these emotions and feelings. Do not dwell on the question for too long, answer sincerely.

1. Desire to achieve the set goal

- Very often
- Often
- Sometimes
- Very rarely

1. Feeling confident in achieving the goal

- Very often
- Often
- Sometimes
- Very rarely

1. Desire to develop a strong character in oneself

- Very often
- Often
- Sometimes
- Very rarely

1. Desire to understand the essence of the process of sports training

- Very often
- Often
- Sometimes
- Very rarely

1. Desire to see through the work started to the end

- Very often
- Often
- Sometimes
- Very rarely

1. Pleasant satisfaction that the work is being done, that time is not wasted

- Very often
- Often
- Sometimes
- Very rarely

1. Desire to understand the conditions for the growth of one's results

- Very often
- Often
- Sometimes
- Very rarely

1. Desire to maximize abilities to achieve success

- Very often
- Often
- Sometimes
- Very rarely

1. Enthusiasm, absorption in work

- Very often
- Often
- Sometimes
- Very rarely

1. Hope for success

- Very often
- Often
- Sometimes
- Very rarely

1. Desire to overcome contradictions in your own reasoning, to bring them into a system

- Very often
- Often
- Sometimes
- Very rarely

1. Desire to resolve your problems

- Very often
- Often
- Sometimes
- Very rarely

1. Hope for achieving very high sports results

- Very often
- Often
- Sometimes
- Very rarely

1. Pleasant fatigue

- Very often
- Often
- Sometimes
- Very rarely

1. Joy of mastering new movements and techniques

- Very often
- Often
- Sometimes
- Very rarely

1. Desire for harmony, smoothness of movements

- Very often
- Often
- Sometimes
- Very rarely

1. Joy of achieving success

- Very often
- Often
- Sometimes
- Very rarely

1. Desire to earn praise

- Very often
- Often
- Sometimes
- Very rarely

1. Sense of satisfaction from improving your physical abilities

- Very often
- Often
- Sometimes
- Very rarely

20. Pleasant satisfaction that you can withstand high physical loads

- Very often
- Often
- Sometimes
- Very rarely

21. Enthusiasm, passion for work

22. Hope for success

23. Sense of satisfaction from overcoming difficulties

24. Desire to gain fame, honor, recognition

25. Effort to resolve contradictions in one's own reasoning, to bring them into a system

26. Desire to solve one's own problems

27. Concern for the fate of one's team

28. Care for someone in the team

29. Pleasant feeling when surveying one's savings, collections, etc.

30. Joy at increasing one's savings

31. Hope for achieving very high sports results

32. Pleasant tiredness

33. Sense of wounded pride and desire for revenge

34. Feeling of superiority

35. Joy of mastering new movements and techniques

36. Aspiration for harmony, smoothness of movements

37. Feeling of gratitude, thankfulness

38. Feeling of sympathy, affection for someone

39. Desire to keep one's sports photographs, posters with one's name, etc., as mementos

40. Pleasant memories of one's sports achievements when reviewing stored newspaper and magazine clippings (videos)

41. Joy of achieving success

42. Desire to earn praise

43. Feeling of pride

44. Feeling of satisfaction that you are growing in your own eyes, increasing the value of your personality

45. Feeling of satisfaction in improving one's physical abilities

46. Pleasant satisfaction that you can endure high physical loads

47. Empathy for another's luck and joy

48. Desire to bring joy to others

49. Elevated mood when the received sports equipment matches the level of upcoming competitions

50. Increased interest in competitions where valuable prizes are at stake.

Then the sum of points is calculated separately for each need, based on the point value of the responses: a - 4; b - 3; c - 2; d - 1.

The numbers of responses included in individual motives are as follows:

Need for Achievement: 1, 2, 11, 12, 21, 22, 31, 32, 41, 42.

Need for Competition: 3, 4, 13, 14, 23, 24, 33, 34, 43, 44.

Need for Self-improvement: 5, 6, 15, 16, 25, 26, 35, 36, 45, 46.

Need for Social Interaction: 7, 8, 17, 18, 27, 28, 37, 38, 47, 48.

Need for Recognition: 9, 10, 19, 20, 29, 30, 39, 40, 49, 50.

The scale for assessing the expression of individual motives in sports activities consists of 4 levels:

Elevated motivation – 30 – 40 points.

Optimal motivation – 28 - 33 points.

Lowered motivation - 19 – 27 points.

Low motivation – 10 – 18 points.

**(3) Endurance test**

Is a Russian adaptation of the English questionnaire Hardiness Survey, developed by American psychologist Salvatore Maddi in 1984. The original adaptation into Russian was done by D.A. Leontiev and E.I. Rasskazova, which was then shortened and subjected to re-validation by E.N. Osin and E.I. Rasskazova. The shortened version of the questionnaire was translated and validated in Russian by M.V. Alfimova and V.E. Golimbet.

*Instruction*. Please answer several questions about yourself. Choose the answer that best reflects your opinion. There are no right or wrong answers here, as only your opinion matters. Please work at a steady pace without dwelling too long on the answers. Work through the questions sequentially without skipping any.

| **Statement** | **No** | **Rather no** | **Rather yes** | **Yes** |
| --- | --- | --- | --- | --- |
| 1. I’m often not sure of my own decisions |  |  |  |  |
| 2. Sometimes I feel like nobody cares about me |  |  |  |  |
| 3. Often, even after a good night’s sleep, I can hardly get myself out of bed |  |  |  |  |
| 4. I’m always busy and I like it |  |  |  |  |
| 5. Often I prefer to "go with the flow" |  |  |  |  |
| 6. I change my plans according to circumstances |  |  |  |  |
| 7. I find it annoying that I have to change my routine |  |  |  |  |
| 8. Unforeseen difficulties sometimes make me very tired |  |  |  |  |
| 9. I always have as much control over the situations as I need |  |  |  |  |
| 10. Sometimes I get so tired that nothing can interest me anymore |  |  |  |  |
| 11. Sometimes everything I do seems useless |  |  |  |  |
| 12. I try to keep up with everything that’s going on around me |  |  |  |  |
| 13. Better a bird in the hand than a crane in the sky |  |  |  |  |
| 14. In the evening I often feel completely broken |  |  |  |  |
| 15. I prefer to set difficult goals and achieve them |  |  |  |  |
| 16. Sometimes I am scared of thoughts about the future. |  |  |  |  |
| 17. I am always confident that I can realize what I have planned in life |  |  |  |  |
| 18. I feel like I’m not living a full life, I’m just playing the part |  |  |  |  |
| 19. It seems to me that if I had had fewer disappointments and misfortunes in the past, it would be easier to live in the world now |  |  |  |  |
| 20. Problems that arise often seem insurmountable to me |  |  |  |  |
| 21. After experiencing defeat, I will try to seek revenge |  |  |  |  |
| 22. I enjoy meeting new people. |  |  |  |  |
| 23. When someone complains that life is boring, it means they simply can't see the interesting things |  |  |  |  |
| 24. I always have something to do. |  |  |  |  |
| 25. I can always influence the outcome of what is happening around me |  |  |  |  |
| 26. I often regret what has already been done |  |  |  |  |
| 27. If a problem requires great effort, I prefer to postpone it until better times |  |  |  |  |
| 28. I find it difficult to get close to other people |  |  |  |  |
| 29. Usually, people around me listen to me attentively |  |  |  |  |
| 30. If I could, I would change a lot in the past |  |  |  |  |
| 31. I often postpone until tomorrow what is difficult to accomplish or what I am unsure of |  |  |  |  |
| 32. It seems to me that life passes me by |  |  |  |  |
| 33. My dreams rarely come true |  |  |  |  |
| 34. Unexpected events give me an interest in life |  |  |  |  |
| 35. Sometimes I feel that all my efforts are in vain |  |  |  |  |
| 36. Sometimes I dream of a calm and measured life |  |  |  |  |
| 37. I lack perseverance to finish what I have started |  |  |  |  |
| 38. Sometimes life seems dull and colorless to me |  |  |  |  |
| 39. I don't have the ability to influence unexpected problems |  |  |  |  |
| 40. People around me underestimate me |  |  |  |  |
| 41. Usually, I enjoy working |  |  |  |  |
| 42. Sometimes I feel like an outsider, even among friends |  |  |  |  |
| 43. Sometimes I have so many problems piled up on me that I just feel helpless |  |  |  |  |
| 44. Friends respect me for perseverance and intransigence |  |  |  |  |
| 45. I willingly take on new ideas |  |  |  |  |

**(4) Multidimensional Personality Self-Realization Questionnaire by S.I. Kudinov**

Test instructions. You are invited to respond to the statements provided in order to identify the features of your self-realization in various areas of life activity.

Respond to each statement as objectively as possible by selecting one of the six answer options.

Remember that the questionnaire does not contain positive or negative statements; each of them may correspond to you to a greater or lesser extent.

Please respond as quickly as possible and choose the answer that comes to mind first.

1 – No

2 – More often no

3 – It varies

4 – More often yes

5 – Yes

6 – Definitely yes

1. The goal of your self-realization is to develop your qualities and abilities.
2. You find it difficult to determine in which sphere you could express yourself and succeed in something.
3. You always try to fully express yourself in everything and everywhere.
4. Most of the time, you have difficulties with taking care of yourself and making any changes within yourself.
5. You feel uplifted when you develop positive qualities in yourself.
6. It is typical for you to immerse yourself in apathy and indifference when you have to work on yourself.
7. You are absolutely sure that personal self-improvement depends solely on the individual.
8. You believe that no effort needs to be made on oneself; a person can achieve spiritual perfection on their own.
9. Your desire for spiritual growth is driven by the desire to contribute to the spiritual and moral development of others.
10. For you, personal self-improvement is associated with the need to achieve inner harmony.
11. Self-realization for you is constant personal growth, daily self-improvement.
12. You believe that self-realization is determined by heredity, and therefore nothing can influence its success.
13. You are convinced that only a high level of spiritual and moral development allows significant achievements in mastering the surrounding world.
14. It seems pointless to you to spend time on your own perfection, as you are surrounded by mediocre people.
15. You are ready to realize all your potential to achieve perfection in the spiritual sphere, but you don't know how to do it.
16. All attempts to change something in yourself are hindered by your uncertainty.
17. From childhood, you purposefully engage in self-improvement every day.
18. The goal of your self-realization is to develop yourself as a person.
19. It doesn't matter to you in which sphere you can realize yourself.
20. You constantly strive to change yourself for the better in spiritual, personal, and physical aspects.
21. You struggle greatly to achieve insignificant results in your self-development.
22. You always experience joy when you realize your own growth.
23. You often experience a mood decline when faced with the prospect of engaging in self-improvement.
24. You believe that every person should exert maximum effort daily to develop themselves as individuals.
25. You agree that exclusively external circumstances and other people entirely determine the moral development of an individual.
26. Your aspiration for constant personal growth is driven by the desire to be helpful to others.
27. Your personal development is dictated by only one goal – to succeed in life.
28. In your opinion, self-realization is primarily the development of positive personal qualities, morality, etc.
29. Self-realization, as you understand it, is a situational manifestation of activity.
30. You think that continuous personal development contributes to achieving success in life.
31. You tend to assume that morally upright people suffer because many do not understand them, or mock and reject them.
32. It seems to you that for complete personal self-improvement, there is a lack of knowledge and experience.
33. Your shyness often hinders your self-realization.
34. You have never felt embarrassed in your life when speaking in front of an unfamiliar audience.
35. In any situation, you strive to manifest yourself in socially beneficial activities to address social issues.
36. You hardly ever take the initiative to express yourself in anything.
37. You constantly strive to utilize all your knowledge and abilities in socially significant events.
38. You almost never show interest in any public actions, movements, parties, or organizations.
39. You derive the greatest satisfaction when engaging in public activities.
40. You are almost always overwhelmed by indifference and apathy if you have to participate in public events (volunteering, rallies, social actions, etc.).
41. You easily handle social work thanks to good self-control.
42. You feel that only professional failures engage in public activities because there is no need to exert any effort on oneself here.
43. In your opinion, self-realization through public activities is often associated with the desire to improve the lives of others.
44. You are not opposed to dedicating yourself to political or other public activities since it brings good dividends.
45. You are confident that a person's self-expression in public activities contributes to their comprehensive development.
46. Self-expression in the public sphere involves flawlessly fulfilling all assignments.
47. Full self-expression in public activities allows one to enrich oneself with new knowledge and experience.
48. You are convinced that expressing your hidden talents in socially beneficial endeavors evokes nothing but envy and resentment from others.
49. The lack of experience and knowledge hinders your self-expression in the social sphere.
50. You know how to succeed in social work, but your stiffness gets in the way.
51. You have never encountered anyone in life who was unpleasant or uninteresting to you.
52. You actively engage in solving socially significant problems.
53. All your aspirations for self-expression are dictated by necessity and obligations.
54. You never miss opportunities to participate in collective endeavors.
55. You are most lazy to fulfill public assignments.
56. You feel exhilarated when faced with the prospect of working in a political party or public charitable organization.
57. Your most negative emotions arise when you are forced to participate in socially significant events.
58. You could achieve great results in any public activity since you always rely solely on yourself and maintain strict control over everything.
59. You believe that public activity is not for you because it requires good organizational skills, which you do not possess.
60. You believe you are ready to dedicate yourself to public activity, even if it means sacrificing your life for the benefit of others.
61. In your opinion, the majority of politicians and public figures pursue selfish goals in their mission, which do not reflect the interests of other people.
62. Full dedication to socially significant activities, in your view, allows one to realize all their intellectual and personal potential.
63. You believe that those who engage in public activities are often life's failures, as it has nothing to do with genuine self-realization.
64. You believe that complete dedication to socially beneficial activities not only leads to success and recognition but also to self-affirmation.
65. Socially beneficial work as a form of self-expression is primitive and unattractive.
66. You are ready to engage in public activities but don't know where to start.
67. You feel embarrassed and anxious when you have to demonstrate your activity publicly.
68. Throughout your life, you have never uttered a single swear word.
69. You exert maximum effort and utilize all your abilities to become a top-class professional.
70. Your self-realization lacks a specific direction and value.
71. You easily engage in any task and fully demonstrate your professional abilities.
72. You apply your knowledge, including professional, only when asked to do so.
73. You experience bright positive emotions when engaged in professional development.
74. Irritability and apathy are characteristic of you when there is a need to acquire new professional knowledge and skills.
75. You are confident that professional mastery depends solely on the individual, not on external circumstances.
76. You believe that achieving the highest professional mastery depends on luck and other people.
77. Your interest in professional development is driven by the need to be useful to other people, the team, and your company.
78. Your desire for professional competence is more closely related to achieving personal moral and material well-being.
79. You believe that true self-realization is only possible in the professional sphere, where a person fully demonstrates their abilities.
80. It seems to you that there is no opportunity for self-expression in the professional sphere.
81. You tend to believe that only pronounced professional self-realization can ensure success in everyone's life.
82. You see no point in constantly improving your professional skills because it often goes unnoticed and does not bring personal satisfaction.
83. In your opinion, professional self-realization is possible only when there is significant experience and knowledge.
84. Your complete self-expression in the professional sphere is hindered by internal constraints, shyness, and difficulty in establishing connections.
85. You can easily persuade anyone of the fallacy of their judgments.
86. The main goal of expressing your inner potential is to become a top-class professional.
87. All your activities are related to fulfilling the dream of occupying a leadership position, regardless of where.
88. You always strive to bring your professional skills and abilities to a high level of mastery.
89. You almost never make efforts for professional self-improvement.
90. You gladly immerse yourself in your professional activities and always strive for excellence.
91. When you think about your profession, you experience a mood decline.
92. You know exactly what you want to achieve in the professional sphere and put in all efforts for it.
93. It seems to you that there is no special need to expend energy and time to improve professionalism since it is not the main thing in a person's life.
94. The main motivation for achieving professionalism for you is the desire to change everything around you, to contribute to the development of the enterprise, city, or country.
95. In your opinion, maximum self-expression in professional activities is always dictated by careerism.
96. In any profession, a person can not only express but also develop their abilities, personal-business, and moral qualities.
97. You believe that success in the professional sphere is in no way related to self-realization.
98. Only full dedication to mastering professional tasks ensures success in life and career.
99. You are confident that professional activity is not the area where a person should or can fully express themselves.
100. You believe that your inability to clearly program your activities hinders you from achieving higher results in professional competence.
101. It seems to you that low self-esteem hinders the development of professional competence.
102. You have never envied other people in anything.
